# An application of solvent and thermal treatment to recover materials from photovoltaic module encapsulated with polyolefin elastomer

**DOI:** 10.1177/0734242X241305092

**Published:** 2024-12-16

**Authors:** Aistis Rapolas Zubas, Inna Pitak, Gintaras Denafas, Egidijus Griškonis, Jolita Kruopienė

**Affiliations:** 1 Institute of Environmental Engineering, Kaunas University of Technology, Kaunas, Lithuania; 2 Department of Environmental Technology, Faculty of Chemical Technology, Kaunas University of Technology, Kaunas, Lithuania; 3 Laboratory of Materials Research and Testing, Lithuanian Energy Institute, Kaunas, Lithuania; 4 Department of Physical and Inorganic Chemistry, Faculty of Chemical Technology, Kaunas University of Technology, Kaunas, Lithuania

**Keywords:** Photovoltaic module, polyolefin elastomer, delamination, encapsulant, recycling, treatment, end-of-life

## Abstract

High-quality recycling of photovoltaic (PV) modules starts with a delamination process. It aims to remove the encapsulation layer between glass and solar cells. Many studies have investigated the delamination of ethylene-vinyl acetate encapsulant, whereas the delamination of polyolefin elastomer (POE) encapsulation in solar modules remains a research gap. This study presents methods of solvent and thermal treatment for the separation of layers in a PV module encapsulated with POE polymer. Various organic compounds were tested for the solvent treatment. The results showed that most of the solvents did not separate the materials. However, with some of them, polymer swelling was achieved. Glycerol was the only solvent capable of separating glass from multi-material laminate. The separated glass does not include contaminants and is therefore suitable for the use as a secondary material. However, the solar cells remained encapsulated in the polymer, thus additional processing is needed to remove it. The time and solvent temperature for glycerol treatment were measured. The thermal treatment was conducted based on the results of thermogravimetric analyses, which determined the degradation of POE under heating conditions. Thermal treatment at 500°C for 1 hour in an air atmosphere was found to be the effective way to detach PV layers. Glass, solar cells and metal ribbons were separated without polymer contamination and are therefore suitable for further use.

## Introduction

Photovoltaic (PV) technology provides an access to clean and affordable energy that plays an important role in the energy transition. It is the fastest-growing renewable energy source, responsible for two-thirds of newly installed renewable electricity sources globally in 2022 (International Energy Agency, 2024). In the same year, the cumulative installed PV capacity reached 1185 gigawatt-peak (Jäger-Waldau, 2023). In order to meet long-term climate goals, the installed PV capacity should reach 80–170 terawatt-peak (TWp) by 2100 (Goldschmidt et al., 2021). However, TWp scale deployment is linked to the high use of materials for PV manufacture (Gervais et al., 2021). Consequently, material recovery from end-of-life (EOL) PV modules becomes a crucial topic (Tao et al., 2020). Technical, economic and legal aspects determine the path chosen for PV waste management (Deng et al., 2022). According to the Waste of Electrical and Electronic Equipment (WEEE) Directive, 80% of the mass of waste PV modules must be reused or recycled in the European Union (European Parliament, 2012). There are several ways to reach the required 80% recycling rate proposed by different research groups (Wang et al., 2022).

The crystalline silicon PV module has the multilayer structure consisting of various materials. One layer called encapsulant consists of a crosslinked polymer. Its crosslinking is achieved through the lamination process of the PV module due to the curing agent in the polymer and the effect of heat and pressure (Landa-Pliquet et al., 2024). When the module becomes waste, the layers have to be separated to recover materials of high quality (Chowdhury et al., 2020). The first step to separate materials from EOL modules is delamination; a process that aims to remove the encapsulation layer that keeps solar cells attached to glass and backsheet (Deng et al., 2019). Various materials are used for PV encapsulation, ethylene-vinyl acetate (EVA) being the most common (Fischer et al., 2023). Its delamination has been discussed in many studies by chemical, thermal and mechanical methods (Wang et al., 2022).

Chemical delamination is based on treatment with organic and inorganic solvents (Li et al., 2022; Min et al., 2023; Pang et al., 2021). Due to the EVA crosslinking in PV, a solvent dissolution of polymer is more complicated compared to non-crosslinked EVA polymer (Deng et al., 2022; Doi et al., 2001). Toluene has been identified as the most effective solvent to swell crosslinked EVA polymer in PV modules (Chitra et al., 2022; Tembo et al., 2021). Use of ultrasound improves the effect of detachment and shortens the residence time (Azeumo et al., 2019). Thermal delamination includes treatment under ambient air (Dobra et al., 2022; Kuczyńska-Łazewska and Klugmann-Radziemska, 2019) or pyrolysis under various conditions (Dias et al., 2017; Wang and Shen, 2022; Xu et al., 2021). During the thermal treatment, the encapsulating polymer is decomposed into volatile compounds (Fiandra et al., 2019). The pyrolysis above 500°C allows to remove >99% EVA from the PV module and separate clean glass from solar cells (Dias et al., 2017). Mechanical delamination can be divided into contact fragmenting, contactless fragmenting, cutting and peeling (Deng et al., 2022). Contact fragmenting treatment can be applied with shredders, hammers, mills or grinders, then connections between layers are broken due to the mechanical force (Azeumo et al., 2019; Dias et al., 2018; Wahman and Surowiak, 2022). After crushing, particles are sorted according to their size. Different fractions contain various material composition (Pagnanelli et al., 2017). Mechanical delamination is the most common method. With mechanical delamination, it is harder to achieve effective separation and/or several additional processing steps may be required to obtain pure materials. Not all polymer material residues can be removed, but still an 80% recycling rate according to WEEE is possible (Pagnanelli et al., 2017; Veolia, 2017).

Delamination research for other types of encapsulation material is still scarce. Polyolefin elastomer (POE) as an encapsulating material in PV modules has proven its resilience and durability through long-term use (Baiamonte et al., 2022). A significant increase in POE encapsulation for PV modules is expected, especially for bifacial products in glass–glass combination and for silicon heterojunction solar cells (Chunduri and Schmela, 2023). EVA remained the mainstream in 2023 with about 70% market share; however, polyolefins are expected to be the dominant polymer in PV encapsulation reaching more than 50% within the next 10 years (Fischer et al., 2023).

To the knowledge of the authors, the only study that examined the delamination of POE-encapsulated PV modules described the use of supercritical CO_2_ (Briand et al., 2023). Neither solvent nor thermal treatments were carried out to separate glass from solar cells. This study aims to determine whether solvent and thermal treatments can be effective for the delamination of PV modules encapsulated with POE polymer.

## Materials and methods

### PV module

**Figure 1.**
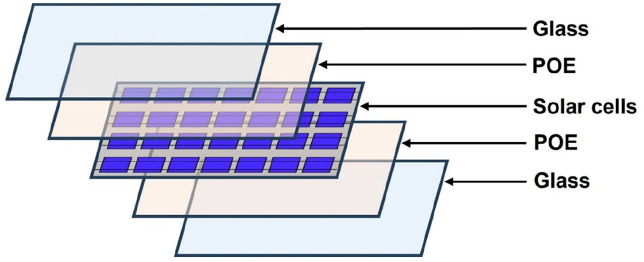
Schematic diagram of the layers in the PV module. PV: photovoltaic.

Solitek Solid Pro 300 W M.60 glass–glass PV module with POE-encapsulation layer was used in this research. The junction box was manually removed from the backside of the PV module before delamination tests. The laminated structure of layers was the same for the whole module area in the following order: tempered glass, encapsulation layer (POE), solar cells, encapsulation layer (POE) and tempered glass. Its schematic diagram is presented in Figure 1.

To obtain samples for tests, the module was cut through the gaps between the solar cells using an angle grinder equipped with a diamond disc. Tempered glass got shattered from both the front and back sides; however, the structure remained stable due to the polymer that kept the laminated layers attached. The resulting samples (~16 × 16 cm) were suitable for thermal treatment in the furnace. Furthermore, the other pieces were cut into ~1.4 × 1.4 cm samples of 2.2–2.8 g for solvent treatment. All samples were fully in conformity to the structure of the module.

### Analyses

A piece was chosen as a representative, and mechanical manual detachment of the glass was conducted on it to obtain the polymer for analysis, which was carried out by Fourier Transform Infrared Spectroscopy (FTIR) method using ALPHA Platinum ATR-FTIR spectrometer in the region of 4000 to 400 cm^‒1^. The results of the analysis were compared with the reference POE described in the literature for the verification of material.

Thermogravimetric (TGA) and differential thermogravimetric (DTG) analyses were performed to identify thermal properties of the encapsulation polymer. The analyses were conducted to determine the temperature at which the encapsulation layer degrades and to understand how the heating conditions affect the polymer’s thermal degradation. The piece of polymer was obtained from the PV module after manual detachment of the glass. Samples of 20 mg were prepared for thermal investigation under nitrogen and air atmospheres. Each sample was placed on the LINSEIS STA PT1600 thermal analyser and heated to 600°C under specified atmosphere at a heating rate of 10 K/min.

### Solvent treatment

Chemical delamination tests were carried out with various volatile organic compounds and high-boiling point organic compounds as solvents: toluene, xylene, dichloromethane, hexane, glycerol, dimethylformamide, diethylene glycol monobutyl ether, polyethylene glycol, melted paraffin wax and a mixture of aliphatic (C_9_–C_20_), aromatic and olefinic hydrocarbons (diesel fuel). The solvents were supplied from various producers. Tests were conducted in laboratory flasks using a sample piece immersed in the solvent by a solid/liquid ratio of 1:15. The sample was immersed in the solvent after the selected temperature was achieved. Temperatures for the reactions were selected according to the type of a solvent, 10°C–30°C below its boiling point. For solvents with the lowest boiling point, a constant temperature of 10°C below their boiling point was applied, whereas for solvents with the highest boiling point, a constant temperature of 30°C below their boiling point was applied. Magnetic stirring was set at 300 rpm to ensure a constant temperature of the solvent. Each chemical delamination test lasted 3 hours. The tests were carried out using the SH-4C digital hotplate magnetic stirrer. A digital power meter was used to count electricity consumption.

### Thermal treatment

The thermal delamination of PV module sample was conducted in accordance with the results of TGAs. The piece of module the size of one solar cell (~16 × 16 cm) was thermally treated under an air atmosphere in the electric muffle furnace (SNOL 8.2/1100) for 1 hour.

## Results

### FTIR analysis

**Figure 2.**
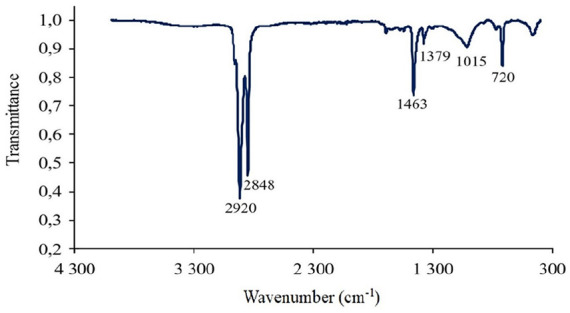
FTIR analysis of the POE encapsulation layer in Solitek Solid Pro 300 W M.60 PV module. FTIR: Fourier Transform Infrared Spectroscopy; PV: photovoltaic; POE: polyolefin elastomer.

FTIR spectra of the POE encapsulation layer in PV module are presented in Figure 2.

FTIR analysis in the region of 4000–400 cm^‒1^ revealed six significant peaks at 2920, 2848, 1463, 1379, 1015 and 720 cm^−1^. To further identify the polymer type, the website: https://openanalysis.org/openspecy, and the materials database were utilized. The analysis results pointed to the presence of polyethylene with a correlation coefficient (*r*) of 0.79. Typical peaks of polyethylene-based materials can be observed at 2920, 2850, 1465, 1370 and 720 cm^−1^. The investigated FTIR spectra were compared with reference FTIR spectra of POE for PV, and significant similarities of peaks were confirmed (Barretta et al., 2021; Fiandra et al., 2024; Lin et al., 2020).

### Solvent treatment

The aim of chemical delamination in recycling PV modules is to dissolve the polymer of the encapsulation layer with the help of a specific solvent. That would allow to separate different layers, in our case – to separate glass from multi-material laminate. We carried out the tests with various organic compounds as solvents. The effect of solvent treatment was determined by visual inspection based on the separation of glass from the multilayer composition of the PV module. The results were classified into one of three options: no separation, swelled POE or detached glass. The results of delamination based on the solvent and the temperature of the test are presented in Table 1.

**Table 1.** Results of solvent delamination on POE encapsulation layer.

Result	Solvent	Temperature (°C)
No separation	Dichloromethane	30
	Diethylene glycol monobutyl ether	200
	Dimethylformamide	140
	Hexane	55
	Polyethylene glycol	185
Swelled POE	Diesel fuel	250
	Melted paraffin wax	340
	Toluene	100
	Xylene	130
Detached glass	Glycerol	270

POE: polyolefin elastomer.

Most of the analysed solvents did not present any physical separation of glass (‘No separation’ in Table 1). With one of them (diethylene glycol monobutyl ether) visual change was observed in the sample of the module (POE became white); however, no separation of layers or physical modification of the structure were identified.

Some of the solvents (‘Swelled POE’ in Table 1) presented polymer swelling. The solvent molecules diffused into the polymer matrix causing the separation of the polymer chains and allowing the solvent to fill the spaces increasing polymer volume (Metze et al., 2023). Although the polymer absorbed a solvent and the volume of the sample increased, the structure of the connected layers remained. Some small pieces of shattered glass from the edges of samples have been separated from the whole structure, but this was mainly due to the mechanical force of the magnetic stirrer. These separated pieces of glass were contaminated with POE residues and require additional treatment to obtain a clean glass cullet.

Glycerol was the only solvent in the study that separated clean glass from the multi-material laminate. The glass cullet was free of polymer residues and therefore suitable for secondary use. Pieces of glass were dried and weighed. The separated glass weighed 1.9 g, accounting for 85% of the mass of the untreated sample (2.2 g). Principles of circular economy can be implemented for further use of recovered material. Separated glass cullet might be an alternative to the use of virgin material for the manufacture of glass.

**Figure 3.**
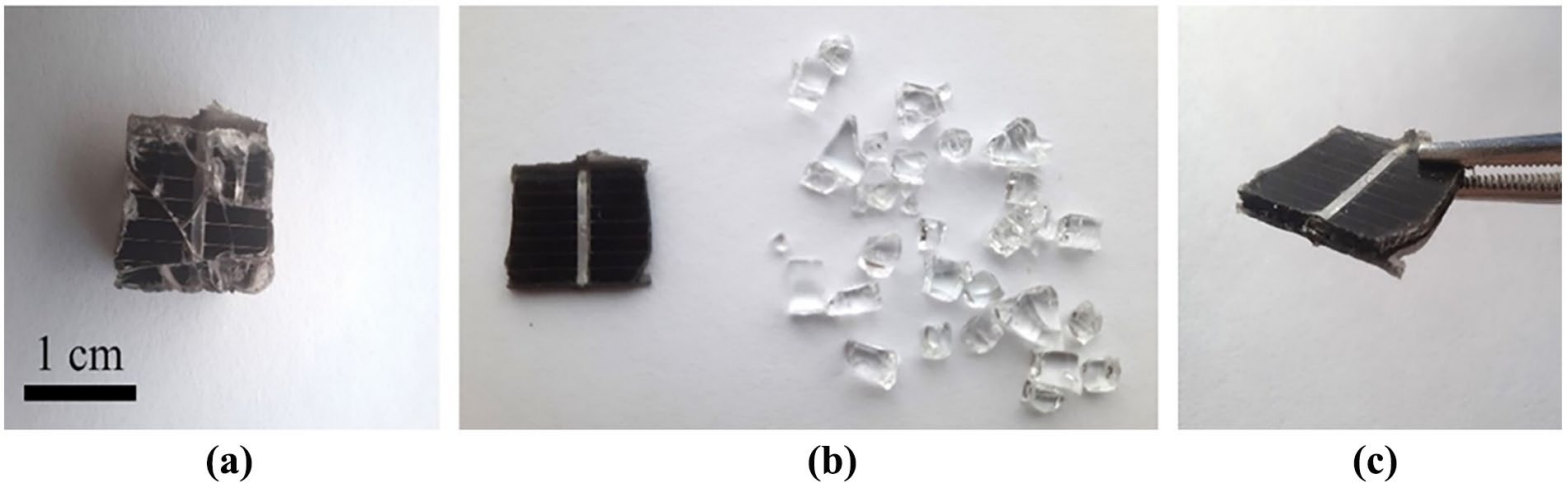
PV sample: (a) before glycerol treatment, (b) encapsulated solar cell and glass after glycerol treatment and (c) encapsulated solar cell after glycerol treatment. PV: photovoltaic.

The links between the glass and POE encapsulation layer were detached during glycerol treatment; however, glycerol was not able to dissolve POE polymer. The separated solar cells remained encapsulated in the polymer layer on both sides. Further processing is required to remove the residual polymer to implement hydrometallurgical methods for metal recovery from the solar cells. Figure 3 shows the sample before solvent treatment and the results of the glycerol treatment of separated glass and encapsulated solar cells. The observed separation of glass from POE-encapsulated PV module creates an interest for additional research. The interaction between glycerol, the glass surface and POE requires further theoretical and practical evaluation. Understanding the nature of these interactions is actual for improving the efficacy of the delamination.

**Figure 4.**
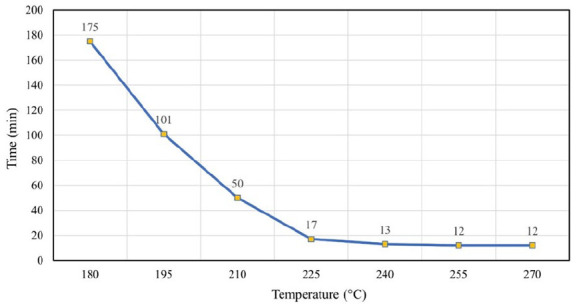
The time of complete detachment of the glass at various temperatures of glycerol.

Additional tests were carried out to determine the temperature and time for glass separation using glycerol as a solvent. The same types of samples and equipment were used as described in ‘Materials and methods’ section. The time of glass separation was determined after the samples were immersed at seven specified temperatures of glycerol. The results are shown in Figure 4.

The increase in temperature by 15°C at lower temperatures (from 180°C to 195°C and from 195°C to 210°C) allowed the separation time to be shortened approximately twice. An almost three times decrease in duration was observed by comparing results at temperatures of 210°C and 225°C. The further increase of solvent temperature did not lead to a significant change in reaction time. About 270°C was the maximum tested temperature because higher temperatures would lead to temperatures near the boiling point of glycerol at 290°C.

The reaction time and temperature affect the amount of energy required for heating and stirring the liquid. Electricity consumption was measured for each test with the digital power metre including the duration of preheating and glass separation. The solvent treatment at 240°C of glycerol for 13 min was determined as the most effective approach in terms of energy use. This treatment consumed 0.31 kWh of electricity in laboratory-scale research. Due to its energy consumption and short process time, the treatment at 240°C of glycerol was found to be the optimal way for the glass separation by the solvent method.

### Thermal approach

**Figure 5.**
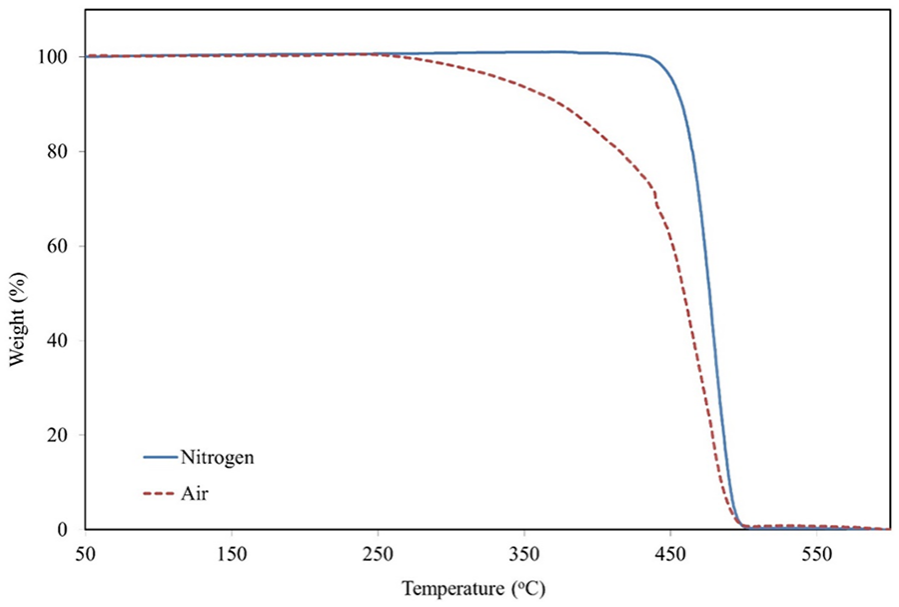
TGA of POE encapsulation layer. TGA: thermogravimetric analysis; POE: polyolefin elastomer.

Thermal properties of the polymer were investigated by TGA and DTG analyses in nitrogen and air atmospheres. The results of TGA are presented in Figure 5.

The weight loss of thermal treatment is more uniform in an air (combustion) than in a nitrogen atmosphere (pyrolysis). Due to the oxygen in air, the oxidation processes started at around 250°C causing thermal degradation of the polymer. The main pyrolysis stage in nitrogen started actively at around 440°C presenting a fast decomposition of high molecular weight compounds into lower molecular weight compounds. Intermediate stages were determined to investigate the thermal degradation of POE. T_5%_ corresponding to the temperatures of 5% weight loss were reached at 338°C in an oxidized atmosphere and 451°C in nitrogen. T_50%_ values were determined at 459°C in air, and 476°C in nitrogen. T_90%_ at 485°C and 490°C in air and nitrogen, respectively.

**Figure 6.**
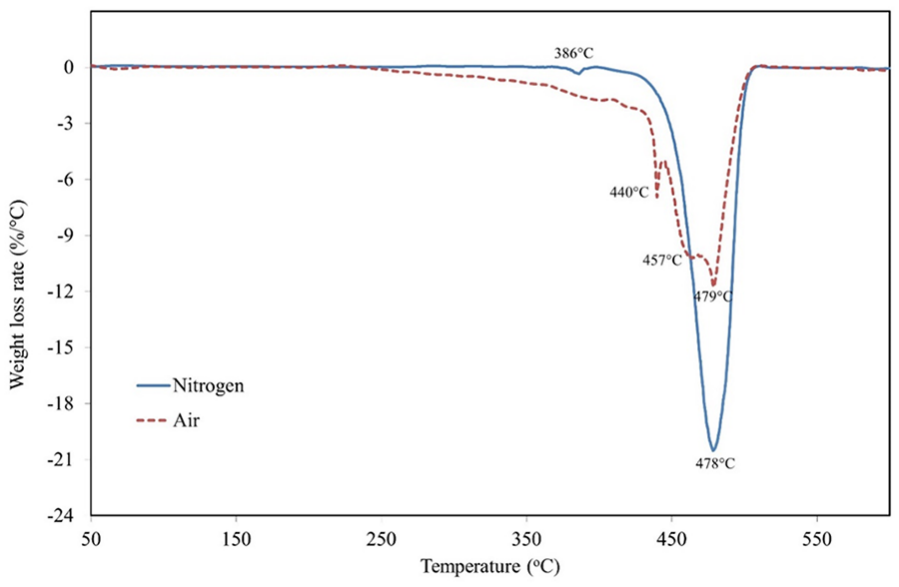
DTG curve of POE encapsulation layer. DTG: differential thermogravimetric; POE: polyolefin elastomer.

DTG analyses were performed to study the thermal stability and decomposition characteristics. Weight loss rate was determined during the thermal treatment of POE encapsulation material. The results in the nitrogen and air atmospheres are presented in Figure 6.

The weight loss rate of thermal treatment in air (combustion) has three main peaks at 440°C, 457°C and 479°C. The weight loss rate of thermal treatment in nitrogen (pyrolysis) has two peaks, a small one at 386°C, and the main one at 478°C. Our thermal analyses of POE encapsulation layer present similarities to the cases described in the literature. Albayrak Hacioglu and Tasdelen (2023) performed TGAs for various compounds in nitrogen atmosphere. The maximum rate degradation temperature for neat POE was determined at 475.8°C. Another study determined the maximum weight loss rate of POE used for PV encapsulation at 474°C in nitrogen atmosphere (Barretta et al., 2021).

**Figure 7.**
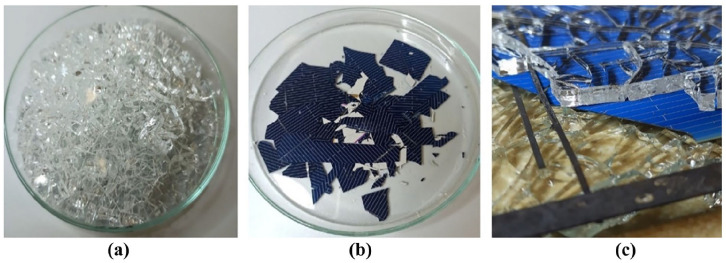
Materials after thermal treatment at 500°C for 1 hour: (a) glass (b) solar cells (c) metal ribbons.

The thermal delamination test was performed based on the results of thermal analyses. A temperature of 500°C was set because it provided more than 99% weight loss by TGA. The duration of 1 hour was chosen as it is sufficient time to ensure that the encapsulant is adequately softened and degraded leading to the complete separation of module layers without significant residues. Thus, the sample of the PV module was thermally treated in an air atmosphere in the electric muffle furnace for 1 hour at 500°C. This treatment completely removed POE polymer and allowed the parts of the PV module to be separated one from another. After thermal delamination, glass, solar cells and metal ribbons were detached. In comparison to the solvent treatment, thermal delamination improved recyclability due to the clean separation of solar cells and metal ribbons. The outcomes of the thermal treatment are shown in Figure 7.

The separated parts were clean of polymer and suitable for further processing. Glass cullet can be used as a feedstock to manufacture new glass products. Contrary to the glycerol treatment, the separated solar cells had no polymer residues. Hydrometallurgical processes can be applied to them for metal leaching from the surface of solar cells. Metal ribbons can be a resource for secondary metal refining.

## Discussion

Our research presented results of solvent and thermal delamination for POE-encapsulated PV module. To investigate a universal delamination way, it is important to analyse differences and similarities between the delamination conditions of EVA- and POE-encapsulated PV modules. Our study demonstrated that glass can be detached from POE-encapsulated PV module by glycerol. The optimal temperature was determined at 240°C due to the lowest energy consumption and the process time of 13 min. In the literature, toluene has been identified as the most effective solvent for the materials separation of EVA-encapsulated PV modules. It provided 30% grade detachment after 1 hour at 25°C, and nearly 74% after 24 hours (Tembo et al., 2021). Our findings indicate that increased solvent temperature shortens process time and improves the detachment efficiency. The same effect was observed for EVA-encapsulated PV modules treated with toluene: detachment grade of 40% was achieved after 30 minutes and 75% after 120 minutes at 60°C; heating toluene to 110°C resulted 100% detachment grade after 30 minutes (Azeumo et al., 2019).

We achieved complete delamination of POE-encapsulated PV module by thermal treatment in air at 500°C for 1 hour based on performed TGAs. Fiandra et al. (2024) examined the thermal properties of EVA, POE and TPO as encapsulating materials for PV modules. Their TGA in nitrogen atmosphere showed similar results to our study. It was found that, unlike EVA, POE has a single stage of degradation and provides better performance without decomposition up to 400°C, making it the most thermally stable encapsulant. The publications on the thermal delamination of EVA-encapsulated PV modules provided findings on process time, temperature and treatment atmosphere. Dobra et al. (2022) reported complete delamination in ambient air after 65 minutes at 500°C, 47 minutes at 550°C and 33 minutes at 600°C. Fiandra et al. (2024) evaluated the efficiency and formation of by-products during thermal delamination at 450°C, 500°C and 600°C on both, nitrogen and oxidized atmospheres, respectively. The process was set for 1 hour, optimal time conditions were not investigated. It was noticed that treatment at 450°C was significantly less efficient than the higher temperatures. The best operating conditions to efficiently degrade the polymer were determined – 500°C in an oxidizing atmosphere. In line with our results, thermal delamination on EVA- and POE-encapsulated PV modules can be implemented under similar conditions. As industrial pyrolysis is carried out on the PV on a large scale, the ability to apply a universal process helps to avoid the polymer detection steps, separated waste streams or various operational conditions.

Further studies are required to optimize the processes and develop delamination. Solvent glycerol treatment left the solar cells encapsulated in polymer, whereas thermal treatment completely dissolved POE encapsulating polymer. Applying thermal treatment only to the encapsulated solar cells could reduce energy consumption by avoiding the need to heat the module’s glass.

Pyrolysis of PV modules in an inert atmosphere was not conducted in this study. However, further research is recommended due to the potential advantages of a simpler and more efficient collection of thermal degradation products. The use of pyrolysis by-products may enhance the overall circularity of PV modules compared to the thermal treatment in an oxidized atmosphere. Gas, oil, aliphatic, aromatic and other compounds are the degradation products of the pyrolysis of polyolefins (Kaminsky, 2016). They can be used for heat generation, or as a raw material for new products. However, the polymer cannot be recovered as a material and used in the same industry. Thus, a direct closed loop is not possible for thermally delaminated encapsulating polymers of PV modules.

An important factor in industrial PV recycling is processing time. Our results for both solvent and thermal treatments indicated longer processing times compared to industrial recycling plants based on mechanical methods. PV dedicated Veolia/PV CYCLE recycling plant includes cutting, grinding, sifting, eddy-current separation and optical sorting achieving over 95% module recycling with a processing time of 1–1.5 min/module (Veolia, 2017). Hot knife technology used to cut the glass from PV modules completes the process in 60 seconds per unit (NPC Incorporated, 2018). Although this technology was applied to EVA-encapsulated PV, it may offer the potential for reducing delamination time for other types of PV modules as well.

## Conclusion

Delamination of POE-encapsulated PV modules is a pressing topic in materials recovery from EOL PV modules. In this work, two methods were investigated for it: solvent and thermal treatment. None of the examined solvents could lead to the complete dissolution of POE polymer in the module. However, the use of glycerol allowed to separate clean glass from multi-material laminate. Due to the high proportional mass of glass, it provided an 85% mass recovery rate from the PV module. Reaction temperature, time and energy consumption were determined for glass separation by glycerol treatment. However, the solar cells remained encapsulated in POE layer and requires further processing to fully remove the polymer for metal extraction from the solar cells. The thermal treatment was carried out based on the results of TGAs. Thermal treatment at 500°C for 1 hour in an air atmosphere was found to be an effective method for the complete POE encapsulant dissolution in the PV module. The outcomes of the treatment were glass, solar cells and metal ribbons without contamination of the polymer. Thermal treatment was determined as a more promising method for material recovery from POE-encapsulated PV modules, as it allowed to detach all PV module parts, increasing recyclability in comparison to solvent treatment.

This study contributes to the approach to delaminate POE-encapsulated PV modules. However, further research is necessary to investigate the effect of alternative compounds for solvent treatment. Thermal treatment in an inert atmosphere is another recommendation for future studies together with the collection and analysis of polymer degradation products.
